# Landmarks, Medical and Surgical

**Published:** 1878-06

**Authors:** 


*Landmarks Medical and Surgical.* By LUTHER HOLDEN, F.R.C.S. Philadelphia: Henry C. Lea. 1878. Buffalo: T. H. Butler.

This little volume of only 128 pages will be found available to the student in acquiring a habit of making the eye and hand work together. Its main objects are to so educate the "touch" upon the normal living body that any deviation may be promptly recognized, and to give the physician such practical directions that a reasonably correct diagnosis may be formed when he is called upon to examine an injury or detect a disease. It is a text-book for clinical use, and as such should be in the hands of every student.

